# Evaluating the Role of Artificial Intelligence in Breast Self-Examination

**DOI:** 10.2196/82550

**Published:** 2025-11-27

**Authors:** Shirish Nayyar

**Affiliations:** 1 General Internal Medicine, King’s College Hospital NHS Foundation Trust, Denmark Hill, London, SE59RS, United Kingdom, 44 7342262402

**Keywords:** breast, cancer, self assessment, AI, DeepSeek, artificial intelligence, breast cancer

## Abstract

This viewpoint explores the role of conversational artificial intelligence (AI) in educating the public on breast self-examination, using an interaction with DeepSeek AI as an example, where 6 AI-generated responses to commonly asked questions were compared with guidelines published by organizations such as the World Health Organization. Although the AI provided clear, accessible, and evidence-aligned responses consistent with professional guidance, limitations included oversimplification and absence of multimedia resources. These findings suggest that AI can support public health education but should be complemented by physician oversight and evidence-based resources for responsible use.

With the growing accessibility of artificial intelligence (AI), the general population is increasingly engaging in conversations with AI chatbots, especially to understand topics related to their body and well-being, hence making it important for the medical community to evaluate and guide the dissemination of related content. This sparked my curiosity to explore one such interaction with DeepSeek AI to generate a general health advice session revolving around breast self-examination.

Breast cancer is a prevalent global health issue, ranking as the second most common cancer in women and the leading cause of cancer-related mortality in women [1]. A breast self-exam, while not a substitute for medical screening, helps one become familiar with how one’s breasts look and feel, making it easier to detect unusual changes, potentially leading to seeking medical aid, early detection, and a better prognosis.

DeepSeek AI is an artificial research company whose aim is to provide cost-efficient but high-performance language models. It offers models at a lower cost than OpenAI’s GPT-4 and is being increasingly used for medical research and development, hence my decision to use this model in a manner aligned with my purpose.

In my recent conversation with DeepSeek AI, I asked the bot 6 commonly asked questions about breast self-examination; the summary of the responses to the same are presented in Table 1.

**Table 1.** Commonly asked questions about breast self-examination and a summary of DeepSeek AI’s responses.

Question	Response
Is self-examination for breast cancer important?	The AI^a^ advised that self-examination plays a key role in early detection but should not replace clinical screenings such as mammograms or professional examinations. It helps individuals become aware of their breast health and detect changes like lumps, dimpling, or discharge between clinical exams. However, it has limitations, as it cannot identify all cancers—some of which may be detected earlier through imaging—and may occasionally cause false alarms due to normal hormonal variations.
How often should I screen myself for breast cancer?	The AI advised that I should perform a self-breast examination once a month to check for any changes, ideally 3‐5 days after my period or on a fixed date if postmenopausal. It explained that doing this monthly helps me track what is normal for my breasts and detect any unusual changes early, such as lumps, dimpling, or nipple discharge. If I find a new hard lump, skin or nipple changes, or persistent swelling or pain, I should see a doctor, as many lumps can be benign. It also reminded me that self-exams are meant to complement, not replace, clinical screenings. I was recommended to follow standard screening guidelines—mammograms typically beginning at age 40‐50 (earlier if high-risk) and clinical breast exams every 1‐3 years in my 20s-30s, then annually after 40. For individuals at higher risk, such as those with a family history of breast cancer or BRCA gene mutations, earlier or more frequent imaging (like magnetic resonance imaging or ultrasound) may be recommended.
How do I screen myself for breast cancer?	The AI outlined that performing a breast self-exam correctly is essential for detecting early changes. It provided a structured, step-by-step guide comprising 3 components: visual check (observing in the mirror for changes in size, dimpling, or nipple appearance), manual check while lying down (using 3 finger pads with varying pressure in circular, vertical, or wedge motions covering the entire breast and armpit), and a shower check (repeating circular motions as soap aids movement). It distinguished between normal findings—soft, slightly lumpy tissue—and warning signs such as hard lumps, skin changes, or bloody discharge. The AI recommended not panicking if a lump is found, as most are benign, but to consult a doctor if the lump is new, hard, or persists beyond 1 menstrual cycle. It concluded by emphasizing that breast self-exams should be done monthly, ideally after the menstrual period, and should complement mammography (recommended from ages 40‐50). For those at high risk, additional imaging such as magnetic resonance imaging or ultrasound may be considered.
Is there more than one way to screen myself for breast cancer?	DeepSeek AI outlined 3 main techniques for performing a breast self-exam, emphasizing that different methods may suit different individuals. The circular (spiral) method involves making small circular motions from the outer edge of the breast toward the nipple, ensuring coverage of the upper chest and underarm for systematic tissue assessment. The vertical strip method entails moving the fingers up and down in vertical lines across the breast, from the collarbone to the bra line and from the side to the underarm and is noted by the American Cancer Society (ACS) as the most thorough approach. The wedge (pie-slice) method involves dividing the breast into “hour-like” sections extending from the nipple outward, allowing a focused examination of each area. For better detection, DeepSeek AI advised performing the exam both while standing and lying down, using soap in the shower for smoother motion, and applying 3 levels of pressure—light, medium, and firm—to assess tissues at varying depths. The optimal time for conducting a breast self-exam is 3‐5 days after menstruation ends. Individuals are encouraged to compare both breasts for symmetry, document any monthly changes, and begin mammograms at the age of 40, or earlier if they are at high risk.
At what age do I start to screen myself for breast cancer?	Breast self-awareness, which involves noticing how one’s breasts normally look and feel, can begin in the teenage years or 20s, while formal monthly self-examinations are typically recommended from age 20. During the teens to 20s, individuals are encouraged to develop breast awareness, with monthly exams being optional unless high-risk. In the 20s to 30s, monthly self-exams may be considered, along with clinical exams every 1 to 3 years, and any changes should be reported promptly. From age 40 onward, self-exams should continue, with annual mammograms added between ages 40 and 50, and high-risk individuals may require additional imaging such as magnetic resonance imaging or ultrasound. Special circumstances include starting screening 10 years earlier than the age at which a family member was diagnosed if high-risk, consulting a doctor during pregnancy or breastfeeding for breast changes, and initiating earlier screening for those with genetic risks such as BRCA mutations or prior radiation exposure. Although self-examinations support early detection, they do not replace clinical evaluations, and screening schedules should be tailored based on individual risk factors.
What findings during self examination should make me see a doctor?	During breast self-exams, certain findings should prompt medical evaluation. Urgent warning signs include a new hard, immovable lump; skin changes such as dimpling, orange-peel texture, or unhealed sores; nipple changes like sudden inversion, bloody discharge, or crusting; unexplained persistent pain in one spot; and sudden asymmetric swelling or distortion. Normal variations include cyclical tenderness or lumpiness before the period, symmetrical texture changes, and small movable lumps that vary with the menstrual cycle. It is advisable to monitor changes through 1 to 2 menstrual cycles, document the size, location, and persistence, and seek prompt evaluation for rapid changes, combined symptoms such as a lump with discharge, or noncyclical symptoms. Most findings are benign, such as cysts or fibroadenomas, but follow-up with clinical examinations or mammograms is essential. High-risk patients may require additional imaging.

a AI: artificial intelligence.

This dialogue highlighted both the potential and the limitations of AI in patient education.

Overall, I felt that the responses to the questions asked were quite comprehensive and detailed, using language that the general population can understand and implement. The responses used words and techniques used by various credible agencies promoting breast self-examination, such as Breastcancer.org and the World Health Organization [2]. The responses also gave an easy-to-understand explanation about the technique of the examination, outlining various alternatives, in line with what is advised by medical professionals and institutions [3]. Additionally, every response stated the fact that breast self-examination was not a substitute for medical examination or investigations, but a complementary practice [4].

Points for further improvement that were clear to me included the provision of links to videos published by credible sites and organizations showing this practice, as 65% of the general population are considered to be visual learners [5]. There is also a risk of oversimplification of the process, especially in cases where the anatomy of breast tissue differs among individuals (such as those with dense breast tissue). There is also not enough emphasis on the fact that breast self-examination is not a substitute for medical examination and screening, which could be reinforced by mentioning certain facts, such as that self-examination, in some studies, is only linked to early detection, but not a reduction in mortality [6].

The utility of AI in medicine also has been raising some ethical and regulatory concerns; for instance, models trained on biased data may reproduce or amplify racial, gender, socioeconomic, or geographic disparities [7]. In addition, chatbots often collect conversational and usage data, bringing about concerns regarding privacy. Methods to mitigate these could be limitation of data collection (collect only what is necessary for the educational task) [8] alongside testing on real-world pilots and external datasets, with integration of these in the bot’s database [9]. Another easy and reliable mitigation strategy would be the use of transparent documentation (data sheets) describing the training data, as well as obtaining clear consent for educational data reuse.

In conclusion, while AI-based conversations can aid in spreading awareness about and reinforcing health behaviors like breast self-screening, they must be complemented by physician oversight, public education campaigns, and accessibility to diagnostic services. Another way to evaluate this could be via the upcoming “evaluator bots,” some of which have been shown to have the same efficacy as human evaluators [10]. Certain facts and percentages must be used to support AI-based responses and stress some important aspects rather than just mentioning them.

I hope this perspective contributes to the ongoing discourse on digital health tools and their responsible integration into medicine.

## Abbreviations

AI: artificial intelligence
